# PickT: A Decision-Making Tool for the Optimal Pickling Process Operation

**DOI:** 10.3390/ma16165567

**Published:** 2023-08-10

**Authors:** Claudia Alice Crișan, Elisabeta Cristina Timiș, Horațiu Vermeșan

**Affiliations:** 1 Department of Environmental Engineering and Sustainable Development Entrepreneurship, Faculty of Materials and Environmental Engineering, Technical University of Cluj-Napoca, 103-105 Muncii Boulevard, 400641 Cluj-Napoca, Romania; claudia.crisan@imadd.utcluj.ro; 2 Department of Chemical Engineering, Faculty of Chemistry and Chemical Engineering, Computer Aided Process Engineering Research Centre, Babeș Bolyai University, 11 Arany János Street, 400028 Cluj-Napoca, Romania

**Keywords:** carbon steel corrosion, pickling bath lifetime, optimum corrosion inhibitor concentration, dynamic mathematical modelling, pickling optimization

## Abstract

This research approaches knowledge gaps related to the pickling process dynamic modelling (the lack of predictability and simplicity of existing models) and answers the practical need for a software tool to facilitate the optimum process operation (by delivering estimations of the optimum corrosion inhibitor addition, optimum pickling bath lifetime, corrosion rate dynamic evolution, and material mass loss). A decision-making tool, PickT, has been developed and verified with the help of measurements from two different pickling experiments, both involving steel in hydrochloric acid. The first round of experiments lasted 336 h (each pickling batch duration was 24 h) and Cetilpyridinium bromide (CPB) was the corrosion inhibitor in additions from 8% to 12%. The collected dataset served for the tool development and first verification. The second round of experiments lasted 10 h (each batch duration was 2 h) and involved metformin hydrochloride (MET) in additions between 3.3 g/L and 10 g/L. This dataset served to test the transferability of PickT to other operating conditions in terms of corrosion inhibitor type, additions, batch duration and pickling bath lifetime magnitude. In both cases PickT results are in accordance with experimental findings. The tool advantages consist of the straightforward applicability, the low amount of field data required for reliable forecasts and the accessibility for untrained professionals from the industry.

## 1. Introduction

Carbon steel has a wide range of applications making it an important material that needs to be produced in large quantities at appropriate quality with the lowest possible costs [1,2]. A key production step serving this purpose is the pickling process (chemical descaling), which helps removing impurities and contaminants from the metal surface [3,4], most often using sulfuric or hydrochloric acid solutions. The use of such highly corrosive acids may lead to problems during pickling (e.g., over corrosion leading to material durability decrease, weight loss and industrial maintenance costs increase). Therefore, various methods have been developed over the years for corrosion detection and decrease [5] and for material protection [6,7,8], among which the use of corrosion inhibitors [9] have been reported to decrease the negative effects of corrosion by more than 20% [10]. Existing research targets a wide range of corrosion inhibitors, with concern towards environment friendly technologies, the use of green inhibitors alone (e.g., *Ircinia strobilina* crude extract [11]; *Amorphophallus paeoniifolius* leaves extract [12]) or in mixtures (e.g., *Sida acuta* and monoethylene glycol [13]; methionine and cetylpyridinium bromide [14]) and the use of drugs as corrosion inhibitors [15]. Such inhibitors are mainly investigated with respect to their discovery and manufacturing process [16], the inhibitor properties [17,18], the inhibitor efficiency [19,20] and the environmental impact [21]. Among green inhibitors tow surfactants, the metformin drug (metformin hydrochloride), C_4_H_11_N_5_.HCl (MET) and cetylpyridinium bromide, C_21_H_38_BrN (CPB), have been reported as effective corrosion inhibitors for carbon steel and copper/copper-iron alloys [14,22,23]. They have been involved in the investigation of corrosion in different corrosive media or in synergistic couples to obtain a higher inhibition rate [24,25,26].

The mathematical modelling of the corrosion phenomenon (with or without the use of inhibitors) is also of significant importance for both industry and academia. Therefore, different mathematical models have been developed to provide quantitative and qualitative information on the corrosion in the context of pickling under different conditions [27,28]. These models could be differentiated as (1) simple non-dynamic, easy to formulate and applicable to certain case studies [29] and (2) very complex comprising multiple parameters (e.g., the immersed surface, pH of the environment, temperature, time of exposure, inhibitor concentration when the case, ion concentration, electric potential, diffusion coefficient, electrode potential, transfer coefficient) [30,31]. Seddiqi et al. [32] represents the corrosion as a galvanic cell and eliminate the chemical potential parameter from the electrochemical equation, to obtain a model capable to predict (within the already conducted experiments time span) the corrosion parameters without further chemical analysis. This model does not include the use of a corrosion inhibitor [32], unlike the work of Gutiérrez et al. [33] that has developed a predictive model focusing on the inhibitor (imidazole and benzimidazole derivates) efficiency and attempting to explain the complex effects of function groups during the inhibition [33]. Both models involve extensive experiments and there is a lack of forecast capability outside the already conducted experiments time span. Such shortcomings tend to be a common point between complex models, while simple models have a lack of wider applicability and forecast capability. All these aspects are important from an industrial point of view, along with the possibility of online monitoring, optimization, and control, as acknowledged by Seddiqi et al. [32]. Moreover, the model ease of use, development cost, labour hand and time spent on experiments must be considered. In this context there is an opportunity, exploited in the present research, to use mathematical modelling to approach the relationship between easily attainable corrosion inhibitor features (e.g., concentration) and the key aspects of the industrial pickling process (e.g., time of bath usage, corrosion rate), while minimizing the effort for the prediction tool development.

The aim of this paper is to develop a decision-making tool for the optimum operation of the industrial pickling process, with respect to the corrosion inhibitor concentration and pickling bath lifetime. The challenge consists in offering an inexpensive technique based on easily attainable parameters without the need to perform complicated experiments. The targeted original contributions and novelty of this work regard the functionality, applicability, and methodological aspects of the software tool: (1) dynamic predictions of the corrosion rate are offered based on easily attainable data; and (2) the optimum pickling bath usage is estimated without the need of further experiments. An additional motivation behind the study is the ambition to deliver a reliable, inexpensive, and easy to use decision support tool to a Romanian steel producer in need to optimize the production flux and use green solutions.

## 2. Materials and Methods

The two main pickling liquors used by the industry are hydrochloric and sulfuric acids. However hydrochloric acid allows faster cleaning rates at lower temperatures and the concentration of the solution is lower than in the case of sulfuric acid [30]. Equations (1)–(3) show the normal evolution of the pickling process in the case of carbon steel and hydrochloric acid while Equation (4) shows the over corrosion of the substrate [34].

(1) $$Fe_{3}O_{4}+8HCl\;\to 2FeCl_{3}+FeCl_{2}+\;4H_{2}O\;$$

(2) $$Fe_{2}O_{3}+6HCl\;\to 2FeCl_{3}+3H_{2}O\;$$

(3) $$FeO\;+\;2HCl\;\to \;FeCl_{2}+H_{2}O\;$$

(4) $$Fe\;+\;2HCl\to FeCl_{2}+H_{2\;}↑\;\;$$

The over-corrosion phenomenon is the main reason for material and economic losses. One of the methods used to control the corrosion is adding inhibitors to the pickling bath. Organic inhibitors containing heterocyclic compounds with polar functional groups (e.g., *N, S, O* and *P*) and conjugated double bonds with different aromatic systems, have been widely used and proven effective over the years [35].

The CPB solution used during the experiments has been generously provided by a local steel manufacturer (Cluj-Napoca, Romania) and the MET drug was acquired from Catena pharmacy (Cluj-Napoca, Romania). Other materials used during the experiments are carbon steel plates purchased from a construction material retailer (Leroy Merilin, Cluj-Napoca, Romania) and concentrated solution of HCl (produced by Sigma Aldrich).

**Figure 1:**
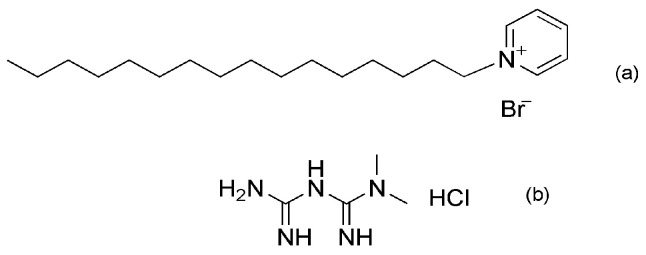
Molecular structure cetylpiridinium bromide (**a**) and metformin hydrochloride (**b**).

CPB and MET are surfactants characterized by the existence of hetero atoms (N, S and O), which are present in both substances tested in this study, as shown in Figure 1. They inhibit corrosion by adsorption on the electrode surface [36].

### 2.1. Experimental Procedure

The method employed for both sets of experiments is detailed hereafter, while particularities of each set of experiments are mentioned in their corresponding sub-sections. Plates of carbon steel of similar shape (50 mm × 50 mm × 1 mm for the first set of experiments and 25 mm × 25 mm × 1 mm for the second set of experiments) were degreased, washed, and initially pickled to be free of any contaminates before starting the experiments. They were put into 50 mL hydrochloric acid solution of industrial making (H_2_O:HCl 1:1, 5.6 M) with different inhibitor concentrations (referred to along the paper as CI concentrations). An additional blank solution, containing no inhibitor, has been employed for comparison purposes. The industrial pickling process mass loss has been illustrated by conducting multiple pickling cycles using the two solutions. After each pickling cycle the plates were removed from the solution, washed with water, dried, and weighted with the help of an analytical balance. The measured mass loss (Δ*m* [g]) values together with the time (t [h]) and the contact surface (S [m^2^]) have been used to calculate the corrosion rate (referred to along the paper as the experimental corrosion rate, *V* [g/m^2^h]) using the widely used Equation (5) [37].

(5) $$V=\frac{\Delta m}{tS}\;\;\;\;\;\left[\frac{g}{m^{2}h}\right]$$

Traditionally, to evaluate the inhibition efficiency of a substance methods such as Tafel polarization or EIS are used. At the same time SEM and/or Raman spectroscopy measurements are carried out for the characterization of the surface, both before and after the corrosion experiment. These methods are shown and explained in the works of Guo et al. [38] and Hossain et al. [39]. However, industrial processes require fast, cheap, and easy enough to understand methods of evaluation on site. Therefore, the weight loss method is the one chosen in this study, to facilitate the further transfer of PickT in industrial sites, as any industrial laboratory features the required equipment.

Particularities of experiments involving CPB. For the first set of experiments the CI concentrations are 8%, 9%, 10%, 11% and 12% CPB. Each concentration has been provided in one solution, except the 12% CI provided in two solutions. The duration of each picking cycle is 24 h and 14 cycles have been conducted over 336 h. The resulting experimental data has been used for the development and calibration of the decision-making tool and for a first verification run (the additional 10% CI experiment).

Particularities of experiments involving MET. For the second set of experiments the CI concentrations are 3.3 g/L, 6.6 g/L and 10 g/L MET. The duration of each picking cycle is 2 h and 5 cycles have been conducted over 10 h. Experimental data has been used for the further testing of PickT’s applicability to different pickling operation conditions.

### 2.2. Computer Tool Develoment Methodology

**Figure 2:**
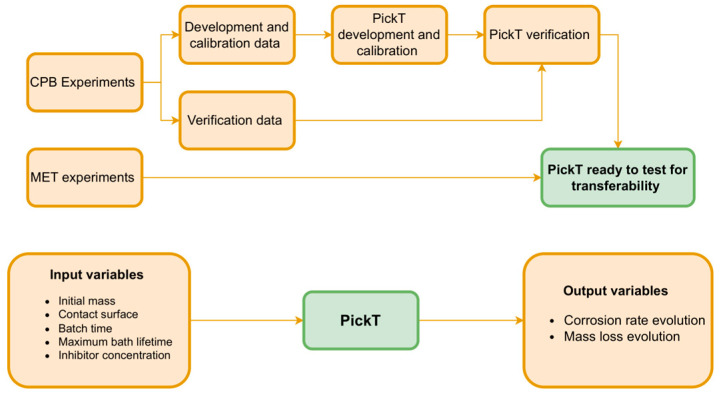
PickT development procedure and architecture.

The first set of experiments, using CPB, has been split in two parts and used for the tool development and first verification with the help of independent data. The second set of experiments, using MET, has been used to test the tool wider applicability and transferability. The PickT development procedure, as well as its input and output variables are illustrated in Figure 2.

Considering the complexity of pickling and its dependence on a lot of parameters [3], our starting hypothesis is that, from a phenomenon point of view the widely used Equation (5) could be improved in the form of Equation (6) by adding additional parameters, to satisfy industrial needs.

(6) $$V_{sim}=X_{1}lg\frac{m_{i}}{S}\;+X_{2}\frac{1}{lg\;t}+X_{3}e^{t/t_{max}}-\;X_{4}e^{CI}$$

The calculated corrosion rate (*V_sim_* [g/m^2^ h], also referred to along the paper as the simulated corrosion rate or corrosion rate forecast) accounts for the presence of the inhibitor via the inhibitor concentration (*CI* [%]); ensures the industrial need of using simple parameters for the prediction via the initial pickled mass of the metal plate (*m_i_* [g]) and the surface of the plate (S [m^2^]); provides dynamic predictions via the batch timing along the entire pickling process (t [h]) and the maximum time in which the pickling bath is going to be used (*t_max_* [h]). X_1–4_ are specific empirical coefficients ensuring the model’s accuracy and suitability for the case study: *X_1_* [1/h] is a shape factor (similar to a first order transformation process constant) related to the mass and surface of the metal plate exposed to pickling; X_2_ [g/m^2^] is a dynamic factor related to the pickling bath duration at each computation step; X_3_ [g/m^2^] is a limitative factor related to the pickling bath duration at each computation step and the maximum considered pickling bath lifetime; and X_4_ [hm^2^/g] accounts for the difference in CI addition.

Material losses are an important factor that needs to be prevented not only for its structural implications but for the economic ones as well. To keep track of this the decision-making tool provides the estimated mass loss vs. the experimental mass loss graph. Further, the calculated mass loss (Δ*m_sim_* [g], also referred to along the paper as the simulated mass loss or mass loss forecast) is obtained with the help of Equation (7) using the corrosion rate calculated with Equation (6) using the already known metal surface and the time passes since the bath started being used.

(7) $$\Delta m_{sim}=V_{sim}\times S\times t$$

To determine the model empirical coefficients an optimization algorithm was developed in MATLAB R2020a. The starting point of the algorithm is the MATLAB R2020a build in function *fmincon*, which uses an objective function that calculates the difference between the two corrosion rates (estimated vs. calculated) at the same point in time based on the correlation coefficient (R^2^) value as criteria for the prediction accuracy. A set of non-linear constraints have been specified in an additional function to eliminate the possibility of having negative unnatural estimated values of the corrosion rate. With the same purpose lower boundaries for the decision variables (X_1_ to X_4_) have been set to ensure a proper correlation between the input variables and the corrosion phenomena. The starting point for the decision variables (X_1_ to X_4_) has been set to zero. Optimum values of the empirical coefficients need to be included in the model for calibration purposes and the model can be further subjected to verification using an additional set of measurements.

The simulation tool results are visual representations of the forecasted corrosion rate and mass loss corresponding to each set of input variables (initial conditions) and comparative representations comprising results corresponding to multiple initial conditions.

## 3. Results

### 3.1. Estimation of Inhibiton Efficency Based on Measurements

The results of measurements shown in Table 1 (using CPB) and Table 2 (using MET) reveal a correlation between the inhibitor concentration and the corrosion rate inhibition. As expected, the biggest mass loss (m [g]) was registered for the Blank sample while the samples immersed in solutions with inhibitor experienced less mass loss. The inhibitor efficiency (*IE* [%]) is estimated using the mass loss with Equation (8), where m is mass loss in samples (i) with inhibitor and (0) without inhibitor [40].

(8) $$IE\%\;=\frac{m_{0}-m_{i}}{m_{0}}\times 100$$

The IE values clearly show that a higher inhibitor concentration corresponds to a lower corrosion rate, which is in accordance with the experimental results and previous studies related to organic corrosion inhibitors [41,42]. The results of CPB experiments (Table 1) indicate 12% as the optimum corrosion inhibitor concentration. This is observed first by looking at the IE and then by looking at the overall mass loss. In the case of the highest concentration the IE is 81.75% for the development sample and 82.90% the verification sample and the overall mass loss is 1.1019 g respectively 1.0326 g, while the Blank sample loses 6.0389 g. A similar behaviour is observed in the case of MET for the experiment involving 10 g/L, corresponding to best IE of 33.41% and an overall mass loss of 0.0748 g, while the blank sample mass loss is 0.1123 g. Even if the inhibition efficiency of MET is low it is important to remark that experiments are suitable for their intended purpose in this research, of testing the transferability and versatility of PickT by using different picking operation conditions compared to the development setup.

### 3.2. Results for CPB during the Developemnt and Verification of PickT

**Figure 3:**
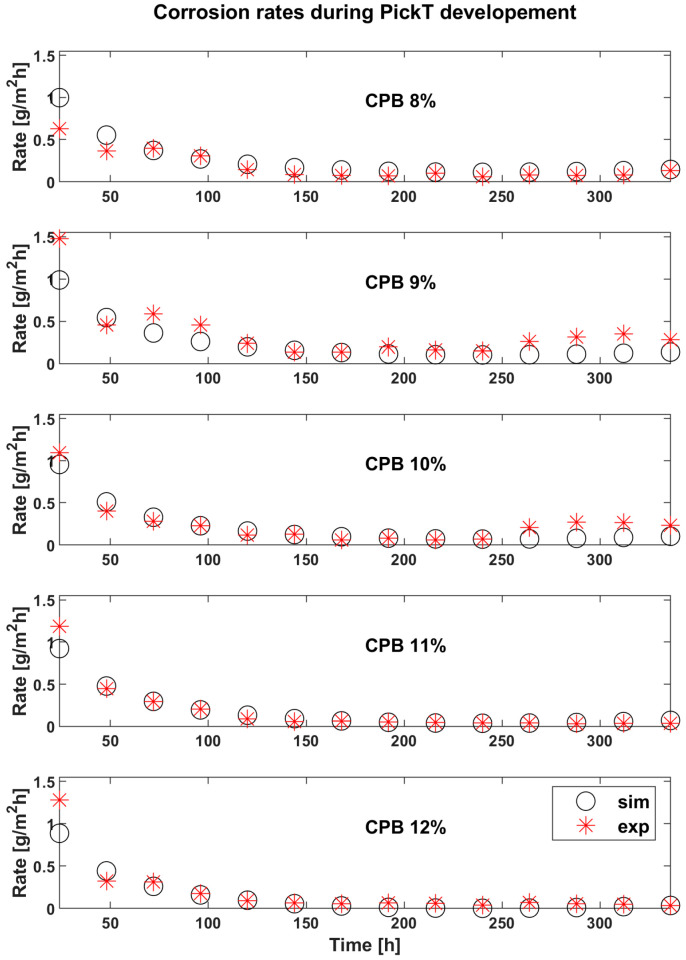
Experimental (exp) results vs. simulations (sim) in time for the corrosion rates corresponding to the tool development runs, employing CPB as corrosion inhibitor.

**Figure 4:**
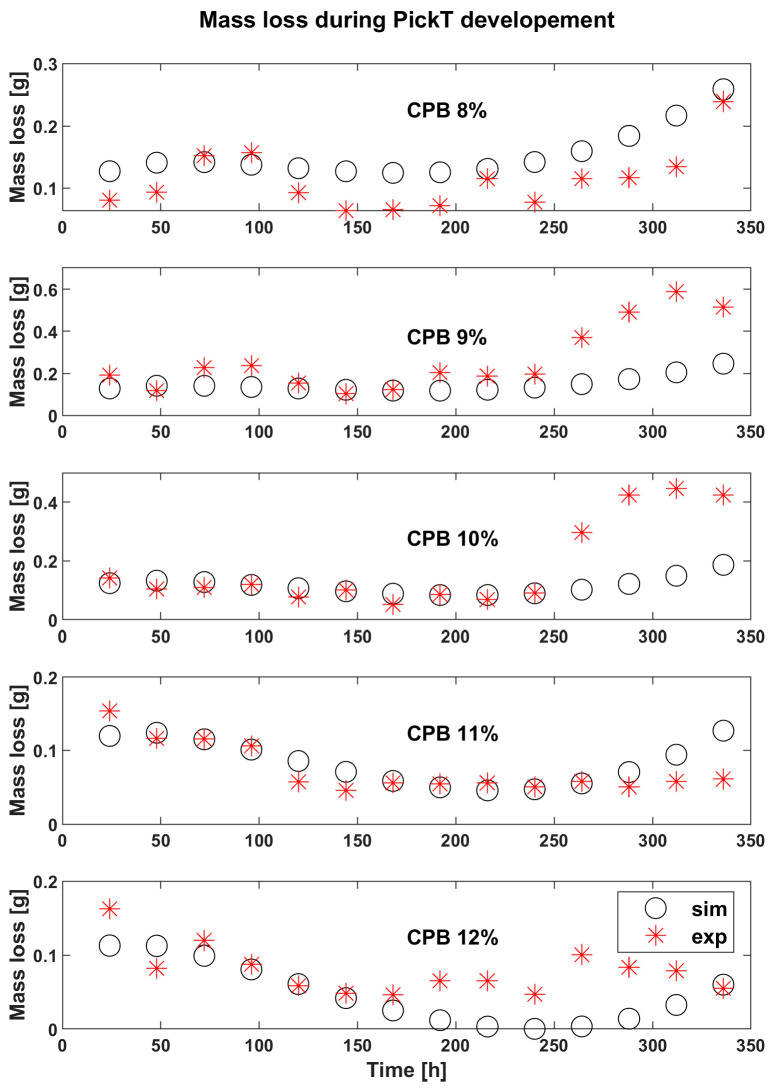
Experimental (exp) results vs. simulations (sim) in time for the mass loss (Mass loss [g]) corresponding to the tool development runs; employing CPB as corrosion inhibitor.

The results of CPB experiments from calibration data are visible in Figure 3 for the corrosion rate and in Figure 4 for the mass loss.

Generally, model development runs involving higher CI concentrations are associated to better prediction accuracy with R^2^ higher than 0.9 for the corrosion rate compared to runs involving lower CI where the R^2^ peaks at 0.89. The R^2^ calculations were done according to Krause et al. [43]. Acceptable predictions of the corrosion rate can be observed without revealing any systematic error patterns or estimation biases in the case of all CI inhibitor concentrations. Calibration runs corresponding to 8% CI and 11% CI exhibit an overestimated forecast mainly towards the end of the simulations (caused by a higher forecast of the corrosion rate), while 9% CI and 10% CI exhibit underestimations. On the other hand, the 12% CI run reveals an underestimated corrosion rate along several pickling batches but ends with a small overestimation.

**Figure 5:**
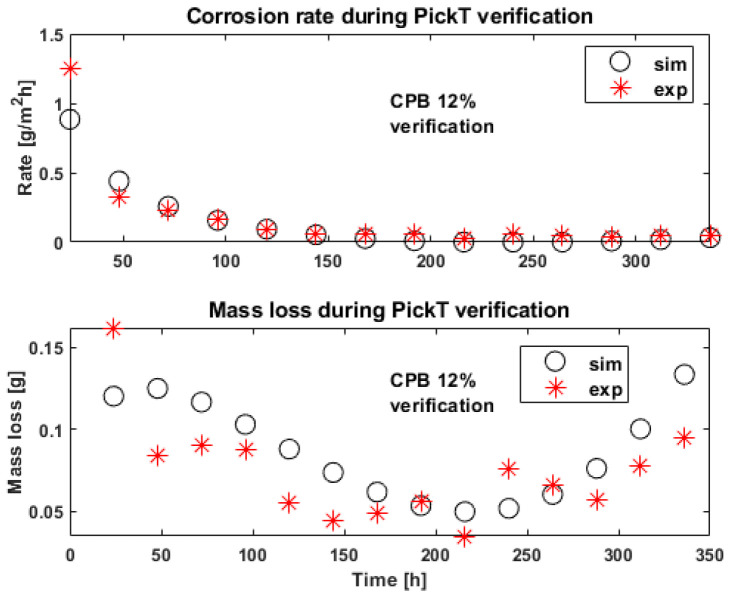
Experimental (exp) results vs. simulations (sim) in time for the corrosion rate and mass loss (Mass loss [g]) corresponding to the tool verification run, employing CPB as corrosion inhibitor.

The experimental data from the pickling bath containing 12% CPB (termed CI 12% verification in Table 1) employed for verification purposes is satisfactory predicted (R^2^ of 0.87), as the simulated corrosion rate and mass loss values follow the trend of values estimated from experimental data (Figure 5). The mass loss overestimation observed towards the end of the pickling process is not an issue, as the industry focus is to correctly predict the optimum length of the pickling process (around the critical point/optimum bath time).

Overall, the model successfully follows the evolution of the phenomena (captured by the experimental values) equally well for calibration and verification runs, showing its applicability within and beyond the experimental data used during development.

### 3.3. The Effectivness of PickT Transferability for MET

**Figure 6:**
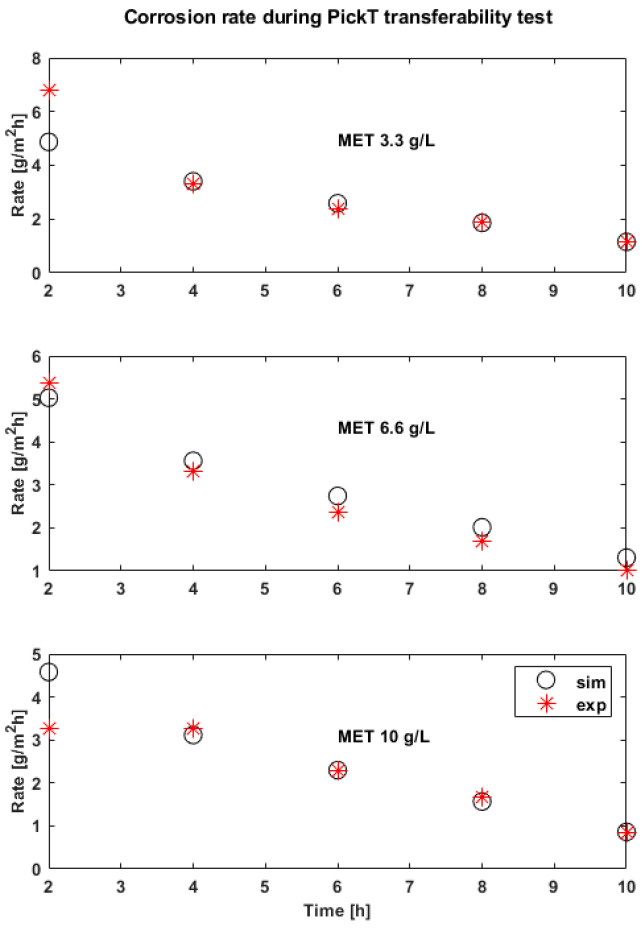
Experimental (exp) results vs. simulations (sim) in time for the corrosion rates corresponding to the tool development runs; employing MET as corrosion inhibitor.

**Figure 7:**
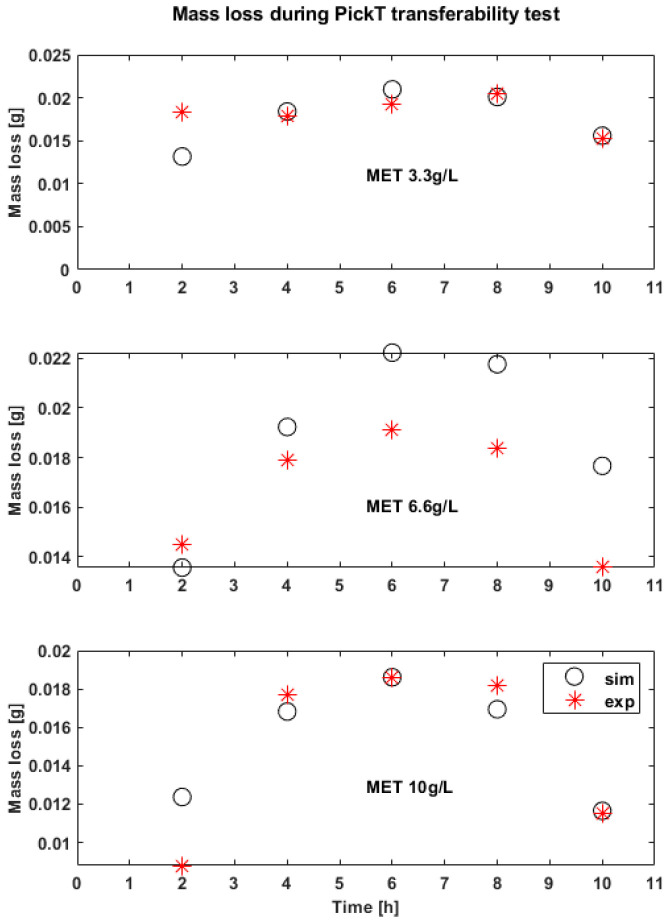
Experimental (exp) results vs. simulations (sim) in time for the mass loss (Mass loss[g]) corresponding to the tool development; employing MET as corrosion inhibitor.

PickT predictions for the additional experiments give good results. The values of R^2^ are 0.80 at the lowest concentration and 0.95 at the highest concentration. As expected for an organic inhibitor the corrosion rate decreases and inhibition efficiency increases with the inhibitor concentration. The trend of experiments is followed by simulation results with overestimations and underestimations of the corrosion rate at the start of the process and more accurate predictions in the later pickling stages (Figure 6 and Figure 7).

## 4. Discussion

### 4.1. Practical Assessment of the Bath Lifetime Using PickT Results

The corrosion rate evolution in Figure 3 and Figure 5 is useful to estimate the optimum pickling bath lifetime for the solutions containing inhibitor, as the appropriate time to change the bath is when the corrosion rate starts increasing, indicating the corrosion inhibitor consumption and the acidic solution exhaustion. The optimum bath time also depends on the CI concentration, as revealed in Figure 3, Figure 4 and Figure 5, where in the time frame between 200 h and 250 h a slight increase in the corrosion rate can be observed across all samples in case of both, the simulated and the experimental values. Comparing the two 12% CI samples focusing on the time frame in which the decision should be made, the same slight increase in corrosion rate is observed however it can be observed that for this concentration the change in solution can happen later after processing a few more batches of product. There are differences when it comes to the beginning of the process where the tool underestimates more in the case of the 12% verification sample and then overestimates the end of the process more for this sample. However, the key time is forecasted correctly.

**Figure 8:**
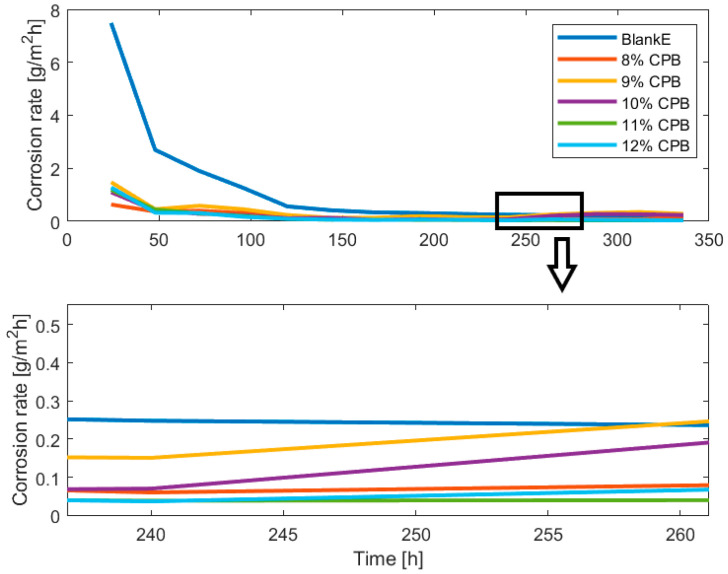
The illustration of corrosion rates dynamics for the estimation of inhibitor efficiency.

A comparison of corrosion rates in case of solutions with corrosion inhibitor against the absence of corrosion inhibitor (BlankE) is presented in Figure 8. In the case of 9% and 10% CPB the corrosion rates start to increase around 240 h while 8%, 11% and 12% CPB solutions continue to have an almost constant evolution and start slightly increasing the corrosion rate only towards the end of the experimental time around T = 300 h. If only the first 3 concentration would be tested the logical conclusion would be that 8% CI performs better than 9% and 10%. Th literature reports that the inhibitor concentration is directly proportional to the inhibitor efficiency, therefore the 2 other concentrations were tested (11%, 12%) with the highest concentration being tested twice for verification of PickT but also to check for the repeatability of the results. Figure 8 shows the moment when the corrosion rate makes an obvious jump and is no longer linear. That being so, the pickling becomes inefficient from a material loss point of view, so the bath needs to be changed soon io the case of those concentrations.

The comparative representation of simulation results for different CI concentrations enables the tool user (e.g., industrial operator) decide which is the optimum CI concentration. Both the results of the experiments and the ones of the simulations reveal that the best inhibitor concentration is 12% in the case of CPB and 10 g/L for MET. The mass loss predictions show that the model does its intended job and confirm that the corrosion rates diagram the tool produces may be used to determine the appropriate time for bath changing.

### 4.2. Discussion in the Context of Existing Literature

Literature reports an increase in inhibitor efficiency with increasing concentration for both inhibitors, thing that PickT is simulating and predicting accordingly. In the case of CPB previous work (Taman and Saleh [22], Zhang et al. [14]) reports similar inhibition efficiency (>70) at higher concentrations. However, the differences between the research in terms of metal substrate (Cu-Fe alloy, mild steel), acidic media (HCl, sulfuric acid (H_2_SO_4_)) or acidic media concentration (HCl 0.5 M, H_2_SO_4_ 0.5 M, HCl 5.6 M) show that this inhibitor is a valuable resource when it comes to corrosion control for industrial applications. MET however has been tested in weak acid media such as 15% HCl [23] or CO_2_-saturated 3.5 wt% NaCl with 340 ppm acetic acid solution [24]. The results show a much higher inhibitor efficiency compared to results in this work. It is suspected that this expired drug is no longer as effective as literature shows once the acid concentration is drastically increased. Even so the PickT predictions remain accurate when it comes to the relation between inhibitor concentration and inhibition efficiency.

According to the authors’ best knowledge existing literature presents no dynamic tools for the prediction of industrial pickling parameters nor CPB and MET have been involved in mathematical modelling studies. Moreover, the mathematical model imbedded in PickT is a simple one compared to what the literature provides when modelling the corrosion phenomenon. It does not require advanced knowledge in areas like molecular chemistry [33] or advanced mathematics [32] which makes it suitable for industrial use. The opportunity to test the PickT wider applicability may be exploited using additional experiments or literature data. The authors did not identify existing pickling experiments conducted to simulate continuous industrial pickling in subsequent batches, nor papers presenting the pickling bath evolution until it becomes spent (exhausted) pickling solution (SPS). Such work may be used for further testing of PickT for other corrosion inhibitors, applying a similar methodology to the one exemplified in this paper in relation to MET. Further work on the matter is considered by the authors employing expired drugs (e.g., Tantum Rosa, Paracetamol) as green corrosion inhibitors.

### 4.3. Industrial Use of PickT: Socio-Environmental and Economical Implications

The local company providing the CPB for this research has a particular interest on the results, especially concerning the bath lifetime, the ease of use, the transferability to a range of corrosion inhibitors, and the accuracy to predict the inhibitor efficiency for different inhibitor concentrations. In response to their needs PickT is simple to use and doesn’t need any special training for the operator of the pickling bath or additional data from further experiments. The tool calibrated for a specific production flux can be used to forecast and support industrial operation across a wide range of parameters (e.g., corrosion inhibitor additions, pickling batch mass, pickling cycle duration). At the same time, from a management point of view the PickT predictions simplify the stages of planning (e.g., chemicals resupply, sludge management and monthly/annual costs), facilitating cost saving at both, the material manufacturer, and the user.

The user costs are usually difficult to quantify and may cover structural failures in the construction sector, pipe breakage in the transportation sector or machine malfunction due to corroded parts [44]. The economic implications for the end users are not as immediate as the ones for the manufacturer, but significant. If the product loses more mass due to the pickling process it will result in structural issues requiring maintenance or a decreased product lifetime. At the same time if the product is not pickled properly the metal surface will present imperfections and impurities that will impact further processing.

Costs covered by the manufacturer will include the cost of corrosion control during manufacturing (e.g., inhibitor consumption, pickling operation, pickling wastewater treatment, specific product quality control), the cost of replacement in case of claims, loss of product proprieties and manufacturing time. The time to change the pickling bath has economic implications on the manufacturing process (e.g., waste amount, costs level, workload) and on the product lifetime. If the bath is changed too soon the inhibitor use is ineffective, increasing the overall inhibitor consumption and the amount of SPS, which may produce an overflow at the wastewater treatment plant or additional costs for the valuable waste recovery (acid) according to the principles of circular economy [45]. Higher SPS output presents economic and ecological concerns since this type of industrial waste is produced in considerable amounts (e.g., only for the steel galvanization are reported 15 to 45 kg SPS /ton [46]). On the other hand, if the bath is changed too late, the metal ions concentration in the bath will increase due to mass loss during pickling, making the regeneration of the pickling bath slower/difficult. PickT allows a company to make a proper schedule for its pickling baths avoiding the overflow or the excessive SPS production while eliminating the additional costs created by the waste of yet usable solution (bath used too short) or unusable solution (bath used too long).

**Figure 9:**
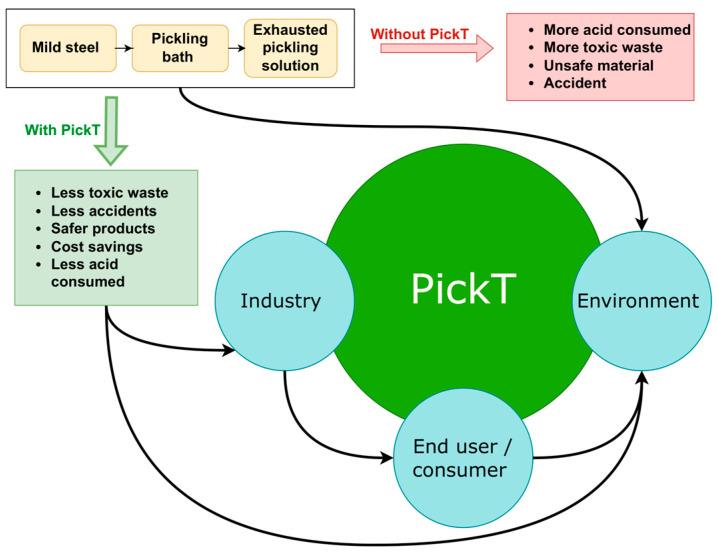
The illustration of PickT impact.

The pickling process has been acknowledged as one of the most impactful steps in industrial metal processing, due to costs, resources consumption and to its environmental impact [47]. A proper SPS management system, to which PickT directly contributes, together with the use of green corrosion inhibitors (e.g., CPB and MET) bring socio-ecological and economic benefits (see Figure 9): ensures safer products (less pitting corrosion, less material loss, and better material integrity), fewer accidents related to structural failures and/or faulty parts, and a better planning of dangerous materials (acidic solutions) consumption and less toxic waste (SPS) to be further processed via treatment or acid recovery techniques (using circular economy tools). Additionally, a lower footprint on water is expected since the pickling solutions are generally 80% water [29] and they need to be neutralized before being introduced into the wastewater treatment circuit. Such benefits directly contribute to the social good since both, the water demand at global level and the water scarcity, are constantly increasing, while the water use could be significantly decreased (by 40% according to [48]) implementing technological improvements. As an example, steel production consumes from 100 to 200 m^3^ of water per ton produced [49].

According to 2021 literature [50] 0.38 million m^3^/year of SPS are produced in EU, while earlier studies mention 0.3 million m^3^/year around 20 years ago [29,45]. In the USA around 5.7 million m^3^/year a production of SPS has been reported [51,52,53]. Assuming the SPS associated processing and management costs around $158.5/m^3^ reported for the same period [52] it is possible to estimate SPS associated costs of $951 million/year only is USA and EU, which at that time produced 34.7% of the total amount of steel at global level (295.3 million metric tons out of 850.1 million metric tons). This amount decreased to 238.3 million metric tons in 2021, while their share decreased to 12.2% (out of 1951.9 million metric tons), as China became a leader in this sector [54]. Assuming similar average production of SPS across this industry and indexing the unit SPS associated costs with an inflation rate of 60% (between 2000 and 2021) the global costs associated to SPS may achieve approximatively $12.5 billion/year. This is part of the total global annual costs associated with corrosion, estimated around $2.5 trillion for 2021 [55], out of which 12.7% (around $317.5 billion) is allocated to production and manufacturing. Following this logic, the SPS associated costs can be estimated at 3.9% of the production and manufacturing costs. In this context the intervention of decision support tools such as PickT may bring significant cost benefits.

## 5. Conclusions

This paper offers an innovative software tool (PickT) to facilitate decision-making in case of industrial pickling. PickT has been developed, verified and its transferability and wider applicability have been tested using two sets of steel pickling experiments involving CPB and MET as corrosion inhibitors in various additions. PickT forecasted the corrosion rate and the weight loss during each pickling cycle, in agreement with the experimental data, with the help of a limited number of easily attainable parameters based on the initial conditions of the process inputs: initial metal surface, corrosion inhibitor concentration, time of immersion (batch duration), and the maximum time of bath usage. The forecasts allow detecting the optimum corrosion inhibitor concentration and the optimum time for acidic bath change. Consequently, PickT eliminates the difficulties of complex models related to the large number of required parameters and additional bath monitoring experiments, while delivering reliable results and addressing industrial needs for simplicity and accuracy. From an industrial operation perspective, it is essential to predict as closely as possible the end time of the pickling bath (due to it becoming inefficient from a material loss point of view), not as much the evolution of its early stages. However, PickT can predict within reasonable margins how the mass and corrosion rate evolve during the process.

## Figures and Tables

**Table 1:** Measured mass loss for experiments using CPB.

Time [h]	Mass of the 7 Plates Immersed in 7 Different Solutions [g]						
	Blank	CI 8%	CI 9%	CI 10%	CI 11%	CI 12%	CI 12% Verification
0	15.1303	11.8791	13.8133	14.0309	14.0601	13.7025	13.9250
24	14.1971	11.7987	13.6220	13.8888	13.9061	13.5397	13.7630
48	13.5248	11.7052	13.5037	13.7848	13.7900	13.4572	13.6788
72	12.8135	11.5528	13.2757	13.6750	13.6746	13.3377	13.5887
96	12.1849	11.3956	13.0388	13.5559	13.5687	13.2501	13.5011
120	11.8349	11.3029	12.8846	13.4797	13.5118	13.1917	13.4458
144	11.5245	11.2397	12.7812	13.3791	13.4666	13.1433	13.4019
168	11.2320	11.1748	12.6584	13.3270	13.4106	13.0970	13.3532
192	10.9193	11.1031	12.4541	13.2417	13.3566	13.0318	13.2977
216	10.6091	10.9884	12.2669	13.1736	13.3009	12.9662	13.2630
240	10.2999	10.9117	12.0713	13.0824	13.2507	12.9191	13.1870
264	9.9774	10.7907	11.7012	12.7862	13.1933	12.8185	13.1210
288	9.6931	10.6805	11.2088	12.3617	13.1432	12.7345	13.0645
312	9.3959	10.5460	10.6209	11.9150	13.0853	12.6557	12.9870
336	9.0914	10.3067	10.1062	11.4896	13.0244	12.6006	12.8924
Overall mass loss [g]	6.0389	1.5724	3.7071	2.5413	1.0357	1.1019	1.0326
*IE%*	-	73.96	38.61	57.91	82.85	81.75	82.90

CI—inhibitor concentration, Blank—solution without inhibitor, IE—inhibitor efficiency.

**Table 2:** Measured mass loss for experiments using MET.

Time [h]	Mass of the 4 Plates Immersed in 4 Different Solutions [g]			
	Blank	CI 3.3 [g/L]	CI 6.6 [g/L]	CI 10 [g/L]
0	4.8685	4.8739	5.0864	5.0260
2	4.8438	4.8555	5.0719	5.0172
4	4.8229	4.8377	5.0540	4.9995
6	4.8005	4.8184	5.0349	4.9809
8	4.7753	4.7979	5.0165	4.9627
10	4.7562	4.7826	5.0029	4.9512
Overall mass loss [g]	0.1133	0.0913	0.0835	0.0747
*IE%*	-	18.70	25.65	33.41

CI—inhibitor concentration, Blank—solution without inhibitor, IE—inhibitor efficiency.

## Data Availability

Data employed for the research is included in the manuscript.
